# Neuromapping olfactory stimulation using magnetoencephalography - visualizing smell, a proof-of-concept study

**DOI:** 10.3389/falgy.2022.1019265

**Published:** 2023-01-09

**Authors:** Rahilla Tarfa, Sophie E. Yu, Omar H. Ahmed, John A. Moore, Ricardo Bruña, Nathalia Velasquez, Alexander J. Poplawsky, Brian A. Coffman, Stella E. Lee

**Affiliations:** ^1^ University of Pittsburgh School of Medicine, Pittsburgh, PA, United States; ^2^ Department of Otolaryngology – Head and Neck Surgery, University of Pittsburgh Medical Center (UPMC), Pittsburgh, PA, United States; ^3^ Department of Otolaryngology – Head & Neck Surgery, Harvard Medical School, Boston, MA, United States; ^4^ Penn Medicine Becker ENT & Allergy, Robbinsville, NJ, United States; ^5^ Department of Radiology, Universidad Complutense de Madrid (UCM), IdISSC, Madrid, Spain; ^6^ Department of Otolaryngology, Cleveland Clinic Florida, Weston, FL, United States; ^7^ Center for Neuroscience, McGowan Institute of Regenerative Medicine, University of Pittsburgh, Pittsburgh, PA, United States; ^8^ Department of Psychiatry, University of Pittsburgh, Pittsburgh, PA, United States; ^9^ Division of Otolaryngology – Head & Neck Surgery, Brigham and Women’s Hospital, Harvard Medical School, Boston, MA, United States

**Keywords:** olfaction, neurological, magnetoencephalagraphy (MEG), electroencephalography (EEG), device

## Abstract

**Importance:**

Currently, clinical assessment of olfaction is largely reliant on subjective methods that require patient participation. The objective method for measuring olfaction, using electroencephalogram (EEG) readings, can be supplemented with the improved temporal resolution of magnetoencephalography (MEG) for olfactory measurement that can delineate cortical and peripheral olfactory loss. MEG provides high temporal and spatial resolution which can enhance our understanding of central olfactory processing compared to using EEG alone.

**Objective:**

To determine the feasibility of building an in-house portable olfactory stimulator paired with electrophysiological neuroimaging technique with MEG to assess olfaction in the clinical setting.

**Design, setting and participants:**

This proof-of-concept study utilized a paired MEG-olfactometer paradigm to assess olfaction in three normosmic participants. We used a two-channel olfactory stimulator to deliver odorants according to a programmed stimulus-rest paradigm. Two synthetic odorants: 2% phenethyl alcohol (rose) and 0.5% amyl acetate (banana) were delivered in increasing increments of time followed by periods of rest. Cortical activity was measured *via* a 306-channel MEG system.

**Main outcomes and measures:**

Primary outcome measure was the relative spectral power for each frequency band, which was contrasted between rest and olfactory stimulation.

**Results:**

Compared to rest, olfactory stimulation produced a 40% increase in relative alpha power within the olfactory cortex bilaterally with both odorants. A 25%–30% increase in relative alpha power occurred in the left orbitofrontal cortex and precentral gyrus with phenethyl alcohol stimulation but not amyl acetate.

**Conclusion and relevance:**

In this proof-of-concept study, we demonstrate the feasibility of olfactory measurement *via* an olfactometer-MEG paradigm. We found that odorant-specific cortical signatures can be identified using this paradigm, setting the basis for further investigation of this system as a prognostic tool for olfactory loss.

## Introduction

The underlying sensorineural networks evoked by olfaction in health and disease in humans is complex and multifaceted. Smell loss is one of the cardinal findings in several prevalent sinonasal diseases including chronic rhinosinusitis (CRS) and most recently has been strongly implicated in patients infected with SARS-CoV-2 (COVID-19). Currently frequently olfaction is measured in the clinical setting by subjective psychophysical testing such as the Sniffin’ Sticks test (1, 2) or the University of Pennsylvania Smell Identification Test (UPSIT) that are validated by multicenter studies (1, 3) but may be subject to patient response bias. Furthermore, as smell recognition is dependent on familiarity with the odor, subjective olfactory testing which rely on scents that are familiar in one cultural context must be adapted for validity in another (4). Finally, in the era of the novel coronavirus (COVID-19), studies have shown that patient’s self-assessment of their olfactory performance may poorly correlate with measured values with subjective testing such as Sniffin’ Sticks (5). As the study of olfaction garners more attention, it is increasingly important to have an objective, portable, and accessible system for measuring smell function. An example of an objective system of measuring olfaction, is utilizing electroencephalogram (EEG) readings to assess the brain's response to olfactory stimulation to distinguish between normosomic, hyposmic, and anosmic patients (6). While an EEG olfactogram is capable of measuring olfaction, magnetoencephalography (MEG) adds another sophisticated level of analysis by providing both temporal and spatial information. Only a handful of studies have demonstrated the efficacy of MEG to detect odor-induced changes in the brain (7, 8). MEG primarily detects the magnetic fields induced by intracellular currents in comparison to the electrical signals which are recorded on EEG. The greater degree of temporal resolution from MEG and spatial resolution from EEG enable complementary information for improved source estimation. Lastly, while most clinicians are familiar with EEG and its clinical applications, the utility of MEG in the clinical setting has been limited to research with questions on epilepsy, audiology, pain, presurgical mapping prior to brain tumor resection, and rehabilitation. In this pilot study, we design and implement a portable magnetic resonance imaging (MRI)-safe olfactory stimulator compatible with MEG to non-invasively assess the neural networks that underlie olfaction.

Previous studies examining olfactory processing have focused on the dynamics in various stages of olfaction, such as laterality and differences in odorant processing based on sex (9–12). While these studies have focused on the physiologic cortical and subcortical responses to odor stimulation, very few have conducted comparative studies to examine changes in olfactory pathologies. One such study, examining evoked olfactory brain signals using MEG to compare normosmic patients to those with hyposmia, was done in the context of Parkinson’s disease (PD) (13). Significant differences were detected in overall spectral power in certain frequency bands in bilateral, central, and temporal cortical and subcortical brain regions between control and PD patients (13). As such, their study demonstrated that odorant-induced changes in spectral power are different between normosmic, hyposmic, and possibly anosmic patients (13).

MEG data is commonly superimposed onto MRI to provide a more user-friendly anatomic interface for data interpretation, the combination of which is called magnetic source imaging (MSI). The regions of the brain thought to be involved in odorant processing, and thus the areas of interest for MSI analysis, are the primary olfactory cortex, hippocampus, parahippocampal gyrus, and orbitofrontal cortex (12). Our proof-of-concept study aimed to achieve the following objectives: (1) to assess the feasibility and safety of delivering olfactory stimulation *via* a programmable MEG-safe olfactometer, while capturing the associated neural processing using MSI (MEG/MRI), and (2) to discern differences in cortical activity between conditions of olfactory stimulation and rest. We hypothesized that our portable olfactometer paired with MEG would be able to elicit central odorant processing differences, revealing distinct odorant signatures during olfactory stimulation.

## Materials and methods

### Participants

Three healthy subjects (two males and one female; mean age 30.6) who were verified to be normosmic by UPSIT testing participated in this study. All subjects were nonsmokers and did not have an upper respiratory illness or acute sinusitis at the time of study participation. Two subjects were right-handed, and one was left-handed. The University of Pittsburgh Institutional Review Board (IRB) approved this pilot study.

### Olfactory stimulator

**Figure 1:**
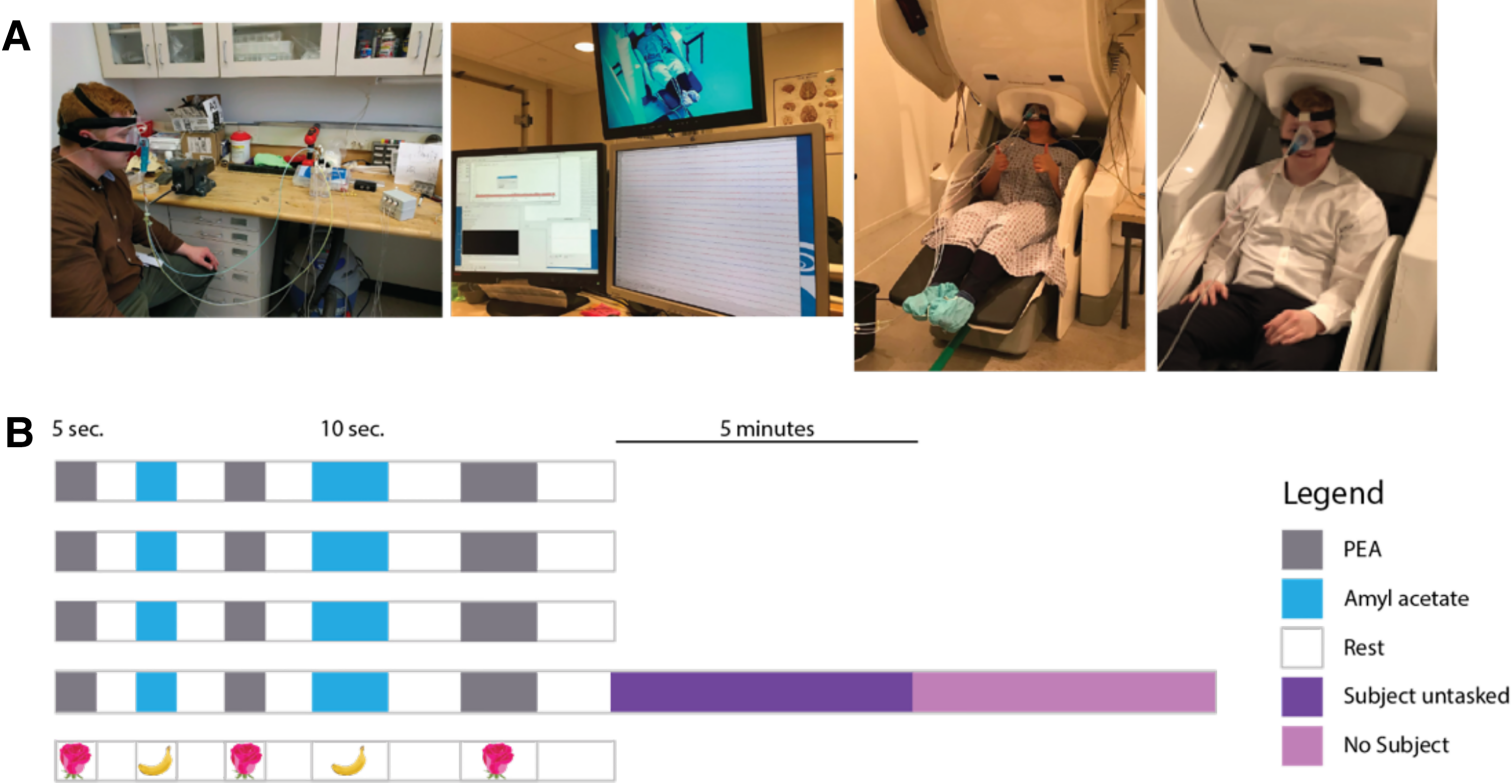
(**A**) Left: depicts the key components of the olfactometer-MEG paradigm. Subject is wearing a CPAP mask that is air sealed. Continuous room air at a constant flow rate is fed into one of three open solenoid valves, triggered by a software program. Air is then fed into one of three glass chambers containing PEA, amyl acetate, or odorless mineral oil. The scented air is then fed into the CPAP mask that is continuously evacuated at a constant flow rate. Middle: 306-channel-whole head MEG. Right: subject wearing CPAP connected to olfactometer and seated with head positioned for the MEG. (**B**) Schematic of software-programmed olfactory stimulus paradigm. Each row corresponds to a “run.” Four runs were performed for each subject with the last run immediately followed by MEG recording of the subject untasked (asked to relax and refrain from making any sustained, voluntary movements) and without the subject present.

A software-programmable, 2-channel, MRI-safe olfactory stimulator was jointly developed by the University of Pittsburgh Division of Sinonasal Disorders and Allergy and the Center for Neuroscience at the University of Pittsburgh McGowan Institute (Figure 1A). The olfactometer was designed to deliver two odorants independently through a solenoid valve-activated mechanism. Both odorants, 0.5% amyl acetate and 2% phenethyl alcohol (PEA), were diluted using odorless mineral oil. Amyl acetate is a synthetic compound used for artificial banana flavoring in food and is well established as a potent trigeminal nerve stimulator. PEA is a synthetic chemical that mimics the scent of rose.

The olfactory stimulator continuously delivered medical room air (21% FiO_2_) at a flow rate of 5 L/min as a conduit to dispense odorants into a nasal CPAP mask that was used to form an airtight seal around the nose. Three 5-volt DC switch-triggered solenoid valves (two valves are always closed while one remains open) (Figure 1A) controlled the flow of both odorants and the odorless mineral oil. The solenoid valves were connected to a BNC switch panel. Each switch was activated in a binary fashion (on/off) by Neurobehavioral Systems Presentation software (Neurobehavioral Systems; Berkeley, CA) using a programmed alternating stimulus-rest schedule (see next section). Continuous room air was fed through one of three Teflon tube lines, each attached to a 300 ml cylindrical glass chamber (Pyrex® round media bottles). One glass chamber housed 50 ml of 0.5% amyl acetate, another housed 50 ml of 2% phenethyl alcohol, and the third housed 50 ml of odorless mineral oil. A wall-mounted flowmeter was used to control the flow rate of room air. Continuous evacuation of contents from the nasal CPAP mask *via* a tygon tube to a wall vacuum was implemented to prevent habituation; the vacuum pressure was kept constant at negative 5 mm/Hg.

### Odorant delivery paradigm

While subjects were seated in the MEG apparatus and recording was active, odorants were delivered according to a specified stimulus-rest cycle schedule. The following “run” (Figure 1B) was performed for each subject four times: 5 s of PEA delivery → 5 s of rest → 5 s of amyl acetate delivery → 5 s of rest → 5 s of PEA delivery → 5 s of rest → 10 s of amyl acetate delivery → 10 s of rest → 10 s of PEA delivery → 10 s of rest. 5 s of PEA delivery followed by 5 s of rest was performed twice. During the session, cortical activity was continuously measured *via* a 306-channel MEG. The solenoid valve allowing airflow into the odorless mineral oil chamber is connected to a 12 V, 0.3-amp DC power supply and is tonically open. The software paradigm delivers 5 V of direct current from the 12 V central solenoid valve, which closes the central control air valve and opens either of the other two odorant delivery valves.

### MEG recording

MEG data were obtained using a 306-channel whole-head MEG system (Elekta Neuromag; Stockholm, Sweden) housed in a magnetically shielded room. The sampling rate was 5,000 Hz and recordings were filtered online with a bandpass from 0 to 1,000 Hz. Four head position indication (HPI) coils were positioned on the subject’s head prior to the recording session to localize each subject’s head relative to the sensor array. Digitization of the subject’s fiducial markings (nasion, bilateral preauricular points, and cranium) was then performed (ISOTRAK; Polhemus, Inc., Colchester, VT) for spatial localization of the emitted electromagnetic signals relative to the subject’s cranial anatomy. Bipolar leads were placed above and below the left eye (vEOG) and lateral to the outer canthi of both eyes (hEOG). Bipolar ECG leads were placed just below the left and right clavicle. Participants were instructed to be relaxed and to minimize sustained volitional movements during the MEG recording. Head movements were minimized as a shelf in the MEG machine maintains the head in a stationary position. Participants were also directed to fixate their vision on a target, an “x” projected onto a blank screen, to reduce eye movement.

Immediately after the four olfactory stimulus-rest runs were complete, recording was performed for 5 min with the subject at rest. Then, recording was performed for 5 min without the subject in the room to identify any artifact external to the subject for subsequent subtraction.

Recordings were offline filtered for interference arising from sources outside of the MEG helmet using the temporal signal space separation method (tSSS) developed by Taulu and Simola (14). A correlation threshold of 0.9 and time window of 10s were used.

### MRI

High resolution MRI images were obtained using a 3-T machine (Figure 1A) (Magnetom; Siemens; Munich, Germany) for all subjects to provide an anatomical reference for the MEG data. T1-weighted sequences were utilized. These images were co-registered with MEG data for each subject using statistical parametric mapping segmentation (SPM version 12, FIL Methods Group, UK) (15).

### Spectral power analysis

Electrophysiological activity in the brain can be seen as a set of overlapping oscillations at different “natural” frequencies, or bands. To study this activity, we have used spectral analysis (Fourier transform) to calculate the oscillatory power in the different bands [alpha (8–12 Hz), beta (13–30 Hz), theta (4–8 Hz), delta (1–4 Hz), and gamma (30–80 Hz)] separately for each olfactory condition and for rest. First, we split each condition into one-second epochs (80 epochs for PEA, 60 for amyl acetate, and 126 for rest). Then, we used Fourier analysis to obtain the amount of power of the oscillatory activity for each frequency value. As Fourier analysis was developed to study infinite signals, we used a smoothing window (tapping window) using Hann's formula, so the signal transits smoothly to zero on the edges. We took the spectrum for each epoch and averaged it to obtain a representative of the spectral information per condition. The total power in each band was calculated by adding the power of all the frequencies following in that band; for example, the total alpha power was calculated as the summation of the oscillatory power at all frequencies between 8 and 12 Hz. Alpha power was compared across conditions using a relative change approach. A threshold of ≥20% change (activity relative to rest) was necessary to be considered significantly different. Comparison of the first 5 s delivery of PEA to the second, to measure a possible habituation effect, was deemed not possible at time of analysis as the time windows were too small for comparison.

## Results

### Demographics

Three participants completed the study. The average UPSIT score was 36.6 (Table 1). All subjects were able to tolerate the study paradigm without any reported adverse events. The average time for completion of MEG testing was 15 min.

**Table 1:** Study participant characteristics.

Demographics	
Sample	(*n* = 3)
Sex	
Male	2 (66.67%)
Female	1 (33.33%)
Age (*M* = 30.6 years of age)	
Subject 1	25.80
Subject 3	32.95
Race	
White	2
Non-hispanic or latino	1 (33%)
Hispanic or latina	1 (33%)
Asian	1 (33%)
Handedness	
Right-handed	2 (66.75%)
Left-handed	1 (33.25%)
UPSIT	
Subject 1	37/40
Subject 2	39/40
Subject 3	35/40

### Results from spectral power analysis

Spectral power analysis was performed for one of the subjects (male, age 25.8, left-handed, Caucasian, UPSIT 37/40). The power spectrum in the classical bands were analyzed, but the larger effect was found in alpha (8–12 Hz). Therefore, results for the alpha band are conveyed below. Nevertheless, the rest of bands are reported in the Supplementary Materials as Supplementary Figures S1–S4.

**Figure 2:**
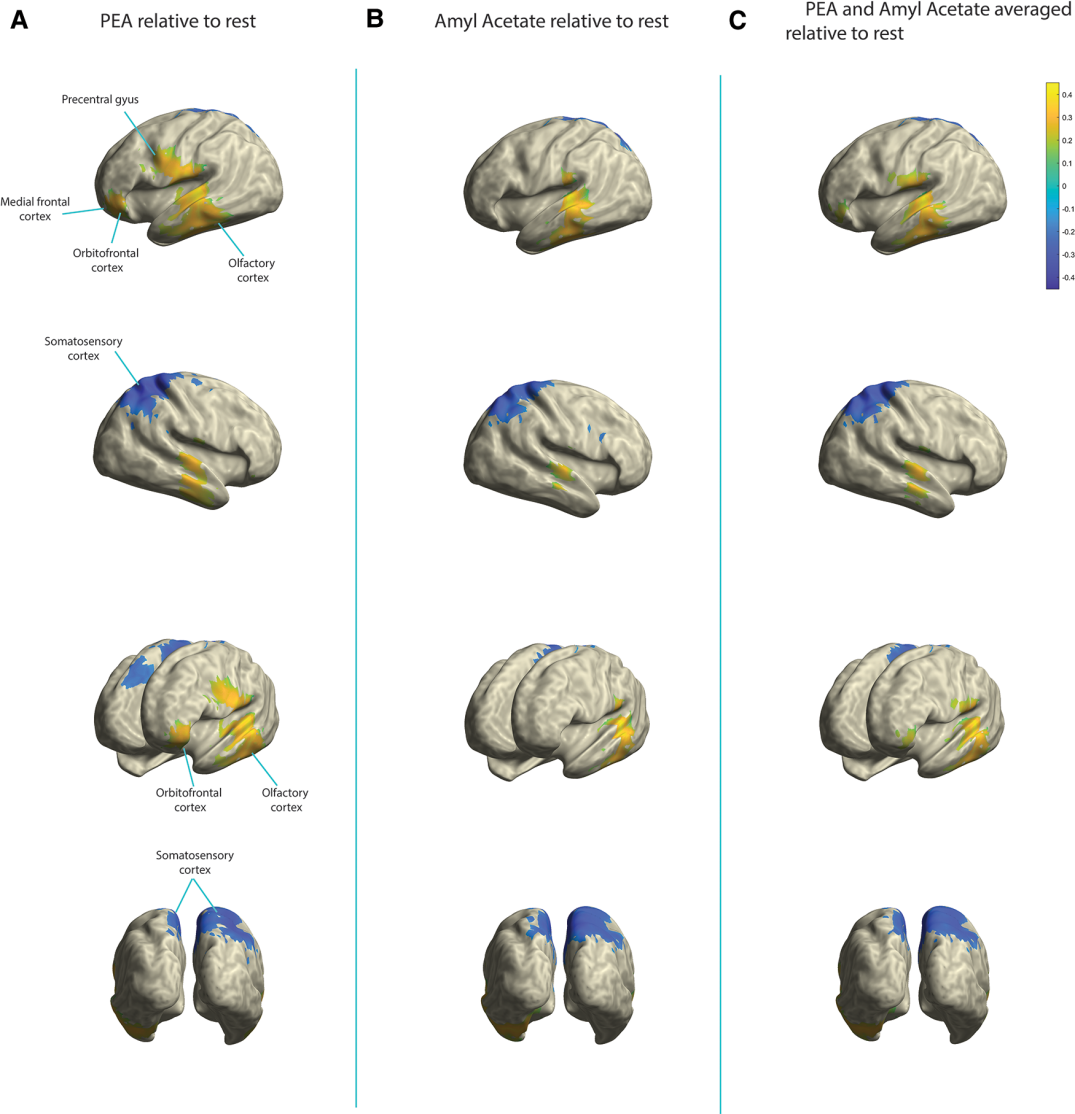
Change in relative alpha power in the olfactory cortex, somatosensory cortex, orbitofrontal cortex, medial frontal cortex, and precentral gyrus. Yellow indicates increases in activity and blue indicates decreases in activity. (**A**) PEA stimulation relative to rest. (**B**) Amyl acetate relative to rest. (**C**) Olfactory stimulation (PEA and amyl acetate signals averaged) relative to rest.

In the alpha band during olfactory stimulation (relative to rest), an approximate 40% increase in relative power was identified in the olfactory cortex bilaterally with both PEA and amyl acetate stimulation (Figure 2). Additionally, an approximate 30% decrease in activity within the somatosensory cortex, right greater than left, was appreciated during olfactory stimulation with both odorants relative to rest. While PEA and amyl acetate generated similar electromagnetic signaling within the olfactory cortex and somatosensory cortex, they also produced different signals in some cortical areas. Relative to rest, an approximate 25%–30% increase in relative alpha power was observed in the left orbitofrontal cortex during PEA stimulation; however, this was not seen with amyl acetate stimulation (<20% detection threshold) (Figure 2). Relative to rest, larger increases (5%–10%) of relative alpha power were observed across a larger expanse of the left medial frontal cortex during PEA stimulation compared to amyl acetate stimulation. Furthermore, in contrast to amyl acetate, larger increases (25%–30%) in alpha power occurred in the left precentral gyrus with PEA stimulation relative to rest.

Individual data files are also available at the following link at “data/segments/” and power results can be found at “data/sources/power_comp/”. Code is available at “script/” https://www.dropbox.com/scl/fo/3u0onblclkdayprxpiwz7/h?dl=0&rlkey=5m6n70okhnytvk9snrgk9aisr.

## Discussion

Using an in-house built portable MRI-compatible olfactory stimulator/MEG system, we were able to elicit olfactory evoked brain networks demonstrating significant changes in the alpha power from superficial cortical brain regions, including the olfactory, somatosensory, medial frontal and orbitofrontal cortices. While we did not observe activity in subcortical structures, this was not unexpected given the limitation of MEG in spatial resolution and algorithms for source estimation (12). Additionally, our preliminary findings are also consistent with a prior study comparing olfaction in healthy patients compared to patients with Parkinson’s disease (PD), showing changes in the alpha band of PD patients (13). It is plausible that the reliable, unique olfactory signatures of odorants that are centrally observed can be utilized to pinpoint long-term central changes in olfactory disorders.

Our olfactometer-MEG paradigm study holds promise as a prognostic tool for hyposmia and anosmia disorders with infectious and inflammatory etiologies. Inflammatory disorders such as chronic rhinosinusitis (CRS) and infectious etiologies *via* viruses like the novel coronavirus (COVID-19) are common causes of olfactory loss. While prior studies have shown peripheral neuroplastic changes in the olfactory bulb following long-term inflammatory olfactory disorders like CRS with nasal polyps (16), corresponding central olfactory changes remain to be elucidated. An objective measure of olfactory function, that combines data from MEG and EEG, would be helpful in diagnosis and management of these patients. Further research is needed to understand the peripheral and central olfactory neuroplasticity changes that underlie these diseases.

Another application of this proof-of-concept study is its role in elucidating olfactory loss underlying neurodegenerative diseases such as Alzheimer’s disease (AD) and Parkinson’s disease. We see future utility in applying this paradigm to (1) investigate it as a possible screening diagnostic for preclinical AD, and (2) develop a more portable and facile prototype device that can be used in the clinical setting to objectively measure olfaction. AD as well as PD are both heralded by olfactory dysfunction (17–21). In fact, olfactory dysfunction is a known predictor of conversion to AD in patients with mild cognitive impairment (20). Olfactory dysfunction has also been associated with more likely and rapid neurological decline in newly diagnosed PD patients (22, 23). Identifying these diseases in their preclinical phase may allow for early intervention which could halt or possibly prevent disease progression (24). While the olfactometer-MEG paradigm has been used to examine differences in MEG activity between normal subjects and those with PD (13), no such study to date has been performed for AD. Additionally, there are currently no validated methods that are approved for diagnosis of preclinical AD.

This study also sets the foundation for development of a MEG and MRI compliant prototype olfactogram device that measures smell electrophysiologically, which can accurately and quickly represent the spectrum of a patient’s olfactory loss.

However, this study must be interpreted within several limitations. The study cohort of three participants was due to limitations of the study IRB and may make the results subject to selection bias. Although the temporal resolution of MEG was utilized in our analysis of frequency bands, the current study did not have enough repetitions to describe the dynamic change of magnetic source imaging over time. Within the methods, leaving 5 s of rest following administration of PEA is too short to analyze the effect of habituation. This study also does not compare the efficacy of the MEG-olfactometer paradigm to other canonical objective systems of measuring olfaction such as the EEG system (25).

In conclusion, the olfactometer-MEG paradigm is a safe, portable, and objective method to study olfaction and its relationship with higher cognitive processing. Data from this study will be used to guide further work in validating this method as a routine, clinical diagnostic modality to objectively evaluate olfaction and a platform to study olfactory dysfunction, including the connection between smell and cortical function.

## Data Availability

The original contributions presented in the study are included in the article/**Supplementary Material**, further inquiries can be directed to the corresponding author.
