# Supplementary material for: Neuromapping olfactory stimulation using magnetoencephalography - visualizing smell, a proof-of-concept study
Source: Front Allergy. 2023 Jan 9;3:1019265. doi: 10.3389/falgy.2022.1019265 (PMC9869273; doi:10.3389/falgy.2022.1019265)
Supplement: Supplementary file 1 [file Datasheet1.pdf]

1 Supplementary Figures

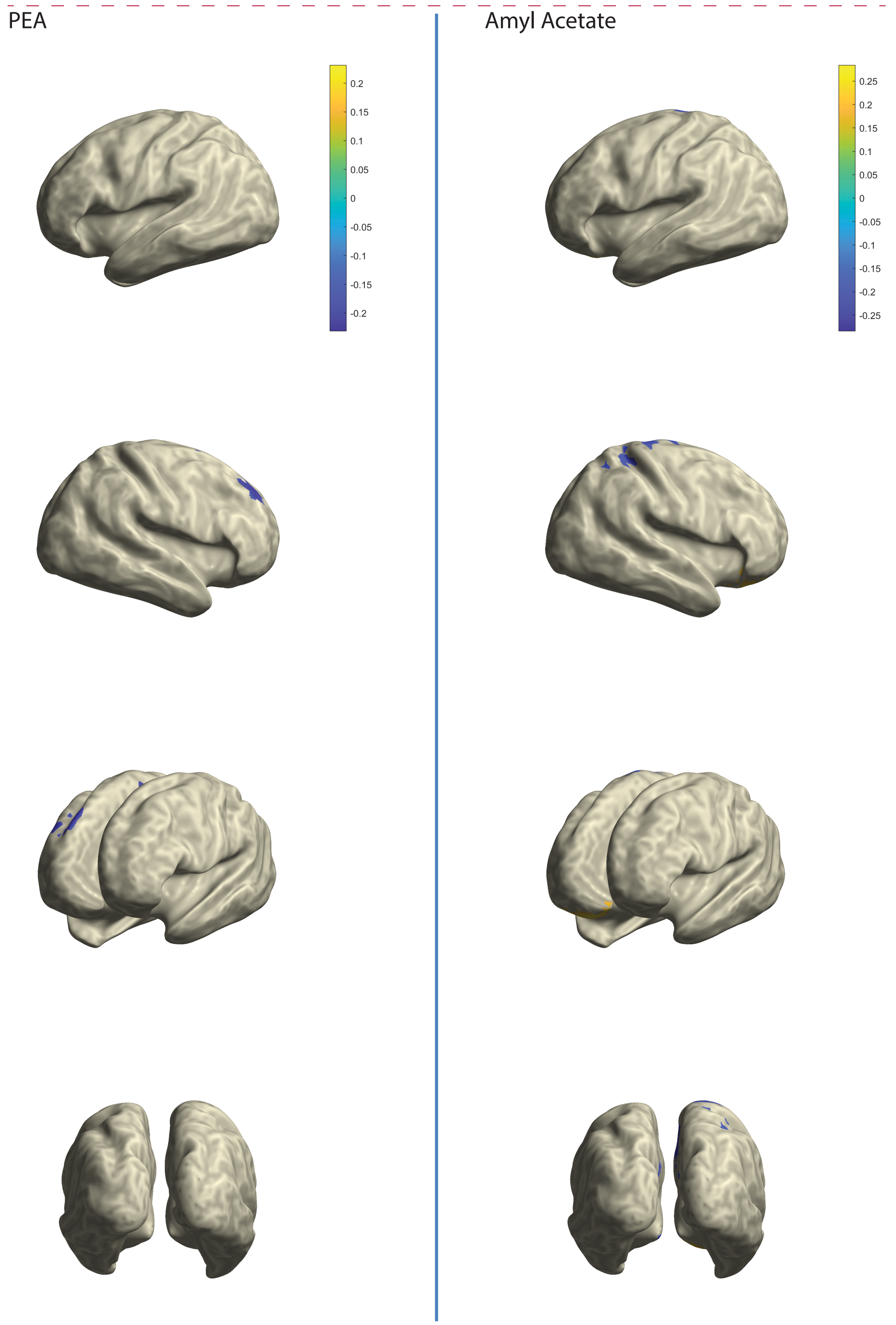

Supplemental Figure 4. Change in relative theta power: olfactory stimulation (PEA and amyl acetate signals averaged) relative to rest. Decimal x 100= % change in relative activity.

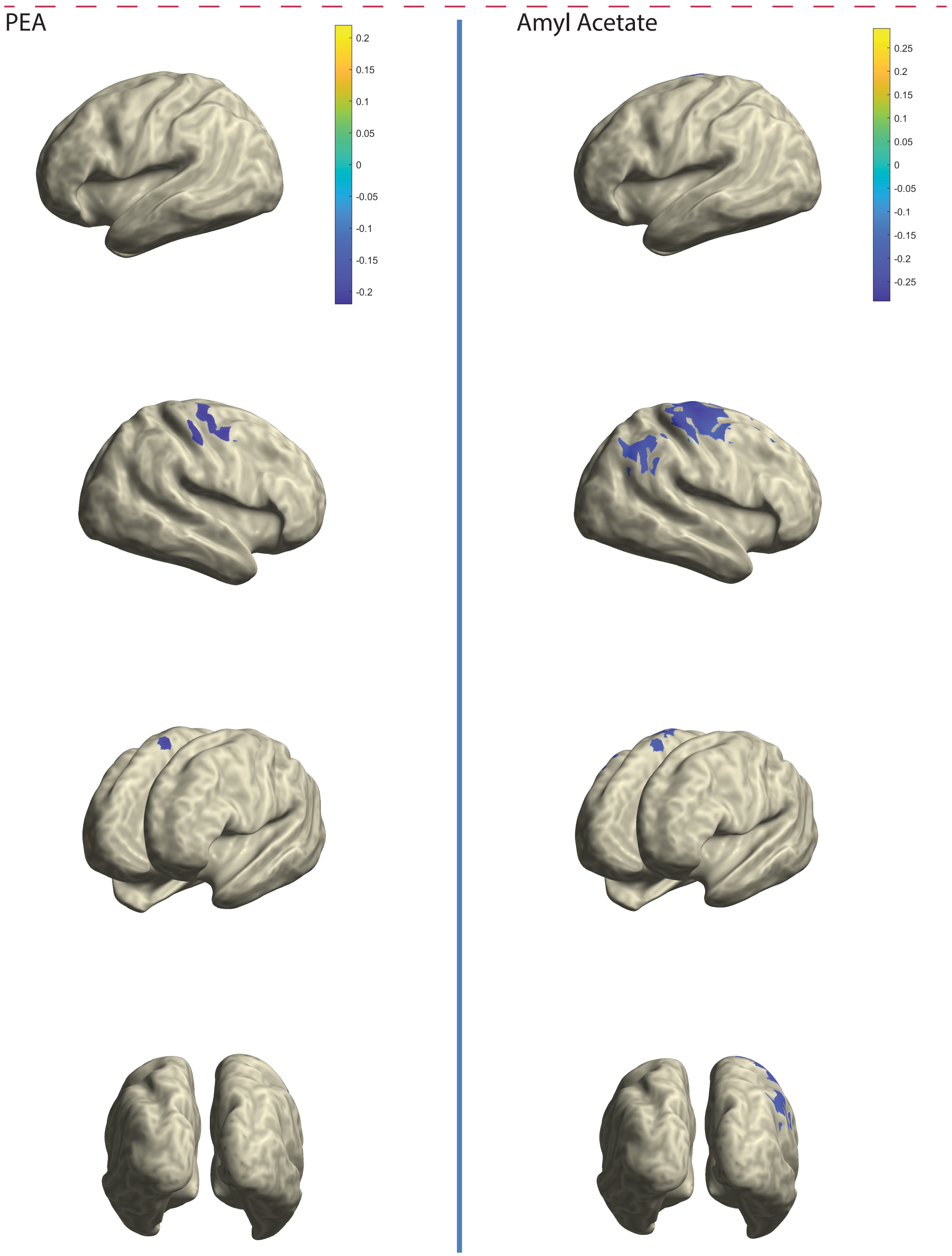

Supplemental Figure 5. Change in relative beta power: olfactory stimulation (PEA and amyl acetate signals averaged) relative to rest. Decimal x 100= % change in relative activity.

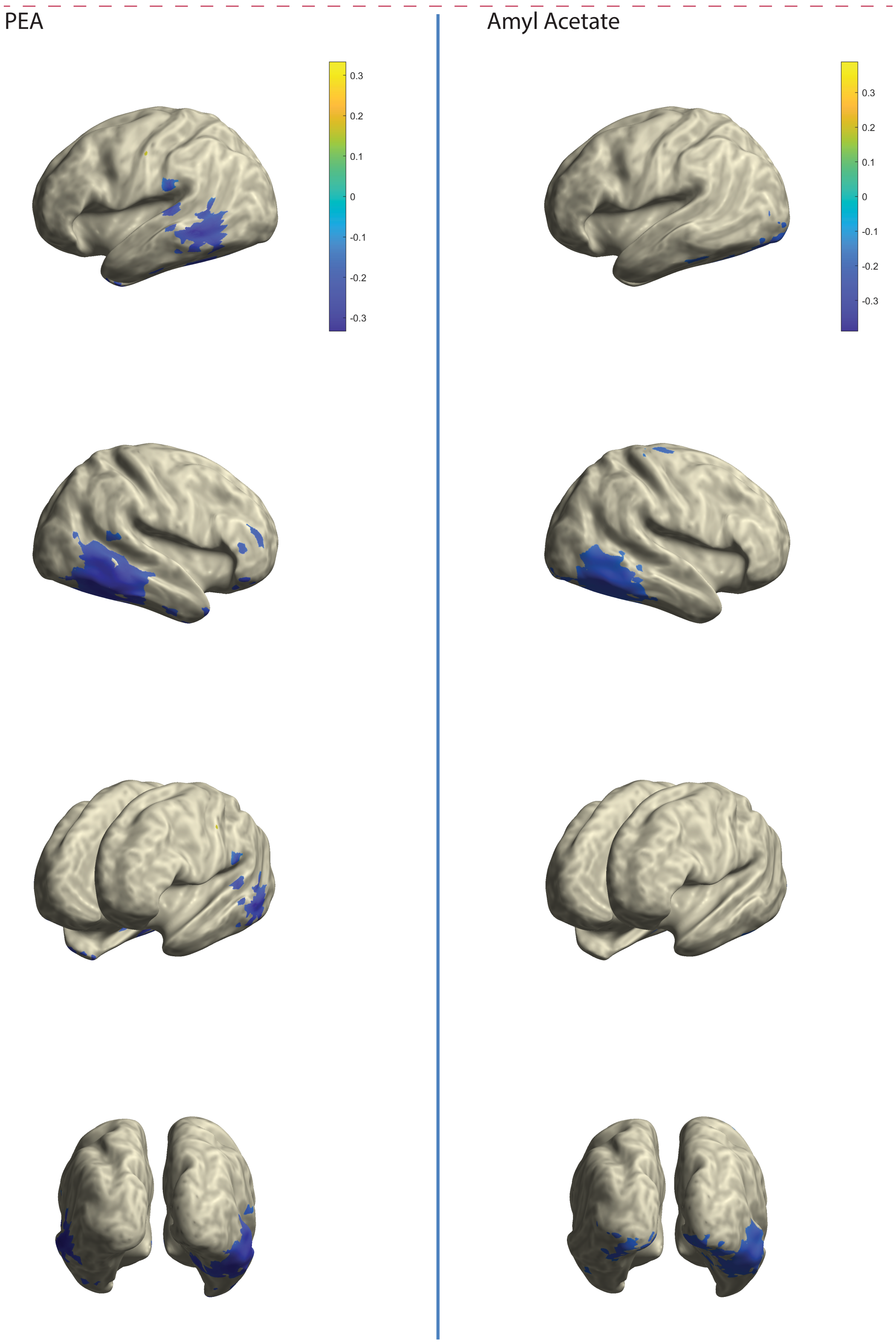

Supplemental Figure 6. Change in relative gamma power: olfactory stimulation (PEA and amyl acetate signals averaged) relative to rest. Decimal x 100= % change in relative activity.
